# Conjunctive Analysis of BSA-Seq and SSR Markers Unveil the Candidate Genes for Resistance to Rice False Smut

**DOI:** 10.3390/biom14010079

**Published:** 2024-01-08

**Authors:** Rongtao Fu, Liyu Zhao, Cheng Chen, Jian Wang, Daihua Lu

**Affiliations:** 1 Institute of Plant Protection, Sichuan Academy of Agricultural Science, Chengdu 610066, China; furongtao@126.com (R.F.);; 2 Key Laboratory of Integrated Pest Management on Crops in Southwest, Ministry of Agriculture, Chengdu 610066, China

**Keywords:** rice false smut, quantitative trait loci, bulk segregant analysis, SSR marker, NB-ARC

## Abstract

Rice false smut (RFS) caused by the fungus *Ustilaginoidea virens* (Cook) leads to serious yield losses in rice. Identification of the gene or quantitative trait loci (QTLs) is crucial to resistance breeding and mitigation of RFS damage. In this study, we crossed a resistant variety, IR77298-14-1-2::IRGC117374-1, with a susceptible *indica cultivar*, 9311, and evaluated recombinant inbred lines in a greenhouse. The genetic analysis showed that the RFS resistance of IR77298-14-1-2::IRGC117374-1 was controlled by multiple recessive loci. We identified a novel QTL, *qRFS12.01*, for RFS resistance in IR77298-14-1-2::IRGC117374-1 by combining bulked segregant analysis with whole genome resequencing (BSA-seq) and simple sequence repeat (SSR) marker mapping approaches. The phenotypic effect of *qRFS12.01* on RFS resistance reached 28.74%, suggesting that SSR markers linked to *qRFS12.01* are valuable for marker-assisted breeding of RFS resistance in rice. The prediction of putative candidate genes within *qRFS12.01* revealed five disease resistance proteins containing NB-ARC domains. In conclusion, our findings provide a new rice chromosome region carrying genes/QTLs for resistance to RFS.

## 1. Introduction

Rice false smut (RFS) is a panicle disease of rice caused by *Ustilaginoidea virens* (Cook) Takahashi [1]. RFS is widely distributed in all rice-growing regions of the world, with the most serious occurrence in Asian rice-growing regions, such as China, Myanmar, India, and Southeast Asian countries [2]. Since the 1980s, RFS has become one of the main rice fungal diseases in China due to the large-scale planting of hybrid rice and the increasing use of nitrogen fertilizer. RFS is not only harmful to the yield and quality of rice, but it also poses a threat to human and animal health due to the toxins present in it. Moreover, it has an inhibitory effect on tubulin in animals [3,4,5]. At present, RFS is controlled mainly by relying on fungicides and adjusting cultivation measures to avoid the disease period, but these measures cannot fundamentally control the occurrence and spread of RFS and cause environmental pollution and fungicide resistance [6,7]. To control the fundamental damage caused by RFS disease, breeding resistant varieties is the most effective measure.

However, there are limited reports on information regarding genes/quantitative trait loci (QTLs) conferring RFS resistance at home and abroad. Xu et al. [8] identified two QTLs, qRFS10 and qRFS12, related to RFS resistance using near-isogenic introgression lines in a Teqing background crossed with Lemont. Li et al. [9] reported that RFS resistance was controlled by two major genes in a polygene mixed genetic model. Andargie et al. [10] identified two RFS-resistant QTLs on chromosome 5 in IR28 using an F_2_ population derived from a cross of rice cultivar IR28 and HXZ. Qiu et al. [11] mapped one major locus on chromosome 1 using an F_2_ population from a cross between Nanjing11 (RFS resistant) and CG3 (RFS susceptible). Han et al. [12] detected five resistance QTLs on chromosomes 2, 4, 8, and 11 using recombinant inbred lines (RILs) derived from the cross of resistant rice landrace MR183-2 with the highly susceptible line 08R2394. Long et al. [13] identified three QTLs conferring RFS resistance using a set of 315 rice accessions from the 3K rice database. Hiremath et al. [14] detected several QTLs on chromosomes 2, 3, 4, 8, 9, and 11 using 125 lines from the global rice diversity panel to perform a genomewide association study (GWAS). Neelam et al. [15] mapped seven QTLs on rice chromosomes 2, 4, 5, 7, and 9 using a RIL population derived from a cross between resistant variety RYT2668 and susceptible variety PR116. Huang et al. [16] detected four QTLs for RFS resistance on rice chromosomes 2, 9, 10, and 11 using the BC1F_2_ and F_2_ populations derived from a hybrid of Xiushui47 and FS159. In addition to the above-cited genes, information on resistance genes, QTLs, and the quantity of resistance sources is limited, and more sources of resistance are urgently needed.

At present, with the development of sequencing technologies and the availability of reference genome sequences of rice, bulked segregant analysis with whole genome resequencing (BSA-seq) is considered an efficient and cost-effective method to rapidly locate genomic regions containing genes or QTLs affecting target traits [17,18]. This method has been successfully used in several plant species, including *Oryza sativa* [18,19], *Medicago sativa* L. [18], *Cucurbita pepo* [20], and *Zea mays* [21]. Compared to traditional linkage mapping, in which offspring are individually genotyped and phenotyped, BSA requires fewer samples for genotyping because only individuals representing the extreme phenotypes of the population need to be identified [22]. In addition, there is no need to develop prior markers in advance for BSA since the selection of whole genome resequencing (WGR) as a BSA genotyping strategy provides an efficient method to estimate genomewide allele frequencies [20]. However, BSA-seq has some disadvantages compared to traditional QTL mapping, such as the inability to detect phenotypic variations or estimates of genetic effects that may deviate from reality.

In the present study, we used a combination of BSA-seq and simple sequence repeat (SSR) markers to detect QTL for RFS resistance in rice. Using a cross between resistant IR77298-14-1-2::IRGC117374-1 (male parent) and susceptible *indica cultivar* ‘9311’ (female parent), our aim was to identify the linkage interval of RFS resistance-related genes in IR77298-14-1-2::IRGC117374-1, and lay a foundation for the fine mapping and gene cloning of resistance-related genes in resistant varieties IR77298-14-1-2::IRGC117374-1. By synthesizing the results of the two QTL mapping approaches, we accurately located the resistance candidate interval and screened candidate resistance genes for RFS.

## 2. Methods and Material

### 2.1. Plant Materials and Pathogen Culture

Resistant rice IR77298-14-1-2::IRGC117374-1 (male parent) [23] and susceptible *indica cultivar* ‘9311’ (female parent) were crossed and produced F_1_ seedings; then, the F_2_ segregation population was produced from F_1_ self-fertilization. IR77298-14-1-2::IRGC117374-1 and 9311 were obtained from the International Rice Research Institute and Changsha Denong Zhengcheng Rice Research Institute, respectively. These rice materials were planted in a controlled greenhouse at the experimental base of the Sichuan Academy of Agricultural Sciences. The whole growth period management was carried out according to conventional cultivation management.

The strongly virulent strains of *U. virens* used for inoculation were preserved by the Plant Protection Institute of Sichuan Academy of Agricultural Sciences, and the strains were PX·D25, GH·DS3, GH·BL, ZZ·Q108, and PJ1 [24]. These strains were grown on potato sucrose agar (PSA) medium, and mycelium disks were placed in potato sucrose broth (PSB). The cultures were incubated at 26 °C on a shaker at 130 rpm for 12 d. The conidia and mycelia of the five strains were collected and mixed for inoculation.

### 2.2. RFS Inoculation and Disease Scoring

At the seventh to eighth stage of panicle development of the parents, F_1_, and F_2_ segregating population, mixed conidial suspension with a concentration of 1 × 10^6^ mL^−1^ was injected into rice panicles. After inoculation, all rice plants were kept at 25/30 °C (night/day), covered with a sunshade net, and automatically sprayed with water for 2 min every 1 h to maintain an environment with 90–95% relative humidity (RH) for 3 d. The number of diseased panicles and diseased grains per panicle was investigated 21 days after inoculation. Each panicle was scored 21 d after inoculation based on a disease rating scale ranging from 0 to 9 (0 = absence of diseased grains per panicle, 1 = 1 diseased grain per panicle, 3 = 2–4 diseased grains per panicle, 5 = 5–8 diseased grains per panicle, 7 = 9–15 diseased grains per panicle, 9 = >16 diseased grains per panicle). The disease index (DI) was used to evaluate the resistance of rice to RFS. The DI was calculated from the disease rating scale using the following formula: DI = ∑ (Number of plants with disease rating × Disease rating)/(Total number of plants × Highest disease rating possible) × 100 [25]. The disease rating scale was estimated by the number of diseased grains per panicle. The evaluation criteria of resistance and susceptibility were as follows: immunization (I): disease index (DI) = 0; high resistance (HR): 0.0 < DI ≤ 5.0; disease resistance (R): 5.0 < DI ≤ 10.0; moderate resistance (MR): 10.0 < DI ≤ 20.0; moderate susceptibility (MS): 20.0 < DI ≤ 40.0; susceptibility (S): 40.0 < DI ≤ 60.0; and high susceptibility (HS): 60.0 < DI ≤ 100.0.

### 2.3. Sample Collection, DNA Extraction and Construction of Segregating Pools

For the SSR markers and BSA-seq analysis, the leaves of the parents and 201 F_2_ plants were collected, labeled, and kept at −80 °C. Genomic DNA extraction was performed using the cetyltrimethylammonium bromide (CTAB) method as modified by Ma et al. [17]. DNA quality and quantity were determined with agarose gel and an Agilent 2100 Bioanalyzer (Agilent Technologies, Santa Clara, CA, USA). Equal amounts of DNA from 20 F_2_ plant lines with extreme resistance (I and HR) and 30 F_2_ plant lines with extreme susceptibility (HS) were mixed to form the resistant (R) and susceptible (S) pools.

### 2.4. Bulk Segregant Analysis Sequencing

We prepared four DNA libraries from the two parents, the R- and S-pools, and performed sequencing. Briefly, the first DNA was randomly fragmented into small pieces of 450 bp by ultrasonication, ligation with the adapters, and purification. Subsequently, 2 × 150 bp double-ended sequencing was performed using the Illumina NovaSeq platform (Shanghai Personalbio Technology Co., Ltd., Shanghai, China). We evaluated and filtered the raw data from sequencing to obtain high-quality reads. Data filtering involved trimming low-quality paired-end reads, reads with more than 50% bases with a Q-score less than 10, poly-N sequences, reads containing an adapter, and those with more than 10% missing bases. The clean reads obtained were aligned to the rice reference genome (Nipponbare) using BWA V7.12-r1039 software) [26]. Subsequently, GATK (Genome Analysis Toolkit) V4.5 software was applied for the variant calling of single nucleotide polymorphisms (SNPs) and small insertion–deletions (Indels) across the parent lines and bulks with the standard filter method [27]. All variants were annotated by ANNOVAR V88 software and combined with reference genome annotation information [28].

Furthermore, we used the Euclidean distance (ED) algorithm to screen candidate regions associated with trait genes [29]. The equation for calculating the ED value based on BSA was as follows:

$$\mathrm{ED}=\sqrt{{\left(Amut-Awt\right)}^{2}+{\left(Tmut-Twt\right)}^{2}{+(Cmut-Cwt)}^{2}+{\left(Gmut-Gwt\right)}^{2}}$$

where each letter (*A*, *T*, *C*, *G*) corresponds to the frequency of its corresponding DNA nucleotide. Before ED calculation, it was necessary to filter the SNPs of the population further. The filtering steps were as follows: (1) preserve the SNP loci that were homozygous but inconsistent with the two parental genotypes; (2) remove loci with a parental depth less than 8×, and (3) remove loci with a F_2_ progeny pool depth less than 8×. The higher the ED value, the higher the linkage strength between the SNP or Indel and the target trait, and the higher the degree of correlation with the target traits. To decrease the background noise caused by the small ED value, we raised the allele frequency ED to 4 powers (ED^4^) [29]. ED^4^ was used for loess fit. The median + 3 standard deviations were used as the threshold, and the region above a threshold was considered the candidate region.

### 2.5. Construction of SSR-Based Genetic Linkage Maps and QTL Analysis

By performing a survey using 871 SSRs, which were evenly distributed across the 12 rice chromosomes, we selected 71 SSR markers representing the polymorphisms between the resistant and susceptible parents (Supplementary Table S1). SSR markers with polymorphisms between parents were selected to detect polymorphisms in the R- and S-pools. SSR amplification was performed in a total volume of 20 µL, containing 17 µL of Goldenstar T6 super mix (TsingKe, Beijing, China), 10 ng of genomic DNA from rice, and 0.5 μM forward and reverse primers. The PCR products were amplified according to the following conditions: initial denaturation at 95 °C for 5 min, followed by 35 cycles of denaturation at 95 °C for 30 s, annealing at 65 °C for 30 s, extension at 72 °C for 30 s, and a final elongation step at 72 °C for 6 min. PCR-amplified products were then separated by 6% acrylamide gel electrophoresis and observed after rapid silver staining [30].

The selected SSR markers that showed clear polymorphism between the R- and S-pools were screened in the F_2_ population and scored as ‘A’, when the allele was similar to the susceptible parent allele; ‘B’, when the allele was similar to the resistant parent allele; ‘H’, when both alleles were presented, and ‘-’, when the allele was absent. Linkage map construction and QTL analysis were carried out using QTL IciMapping V4.0 [31]. The linkage map was created using the MAP function in QTL IciMapping V4.0 software, with a minimum LOD value of 3.0. Genotypic data and phenotypic data were combined to analyze the QTL. QTL analysis was performed using the BIP function in QTL IciMapping. The inclusive composite interval mapping of additive (ICIM-ADD) QTL method with mapping parameters of a walking step of 1.0 cm and a probability of stepwise regression of 0.001 was used for the significance of QTL identification [25]. The threshold value of LOD was set at 3.0 or higher to improve the accuracy of the detected QTL [32]. The identified QTLs were named based on the nomenclature reported by McCough and Doerge [33].

### 2.6. Candidate Gene Identification

QTL regions that identified the overlapping physical locations of BSA-seq and SSR markers were taken as the candidate regions. The genes in the candidate regions were annotated using Gene Ontology (GO), Kyoto Encyclopedia of Genes and Genomes (KEGG), Nonredundant Protein Sequence (NR) database, and rice gene annotation database (http://rice.uga.edu/index.shtml (accessed on 26 June 2023)) to predict their function as candidate genes related to disease resistance.

### 2.7. Expression Analysis of Putative-Resistant Genes by RT-qPCR

To evaluate gene expression, we conducted reverse transcription quantitative PCR (RT-qPCR). Total RNA was isolated from rice panicle tissues of two parents using TRIzol (Aidlab Biotechnologies, Beijing, China) according to the manufacturer’s protocol and then converted to cDNA using a First-Strand cDNA Synthesis Kit (Sangon Biotech, Co., Ltd., Shanghai, China). The qRT-PCR experiments were carried out using a qTower3G Real-Time PCR System (Analytik Jena AG, Jena, Germany) according to the manufacturer’s instructions. The PCR system contained 2 μL of cDNA, 0.4 μL of each specific primer, 10 μL SYBR Premix ExTaqTM (Takara, Dalian, China), and 0.4 μL ROX reference dye. The primer sequences of the genes are listed in Supplementary Table S2. Four biological replicates were used for each treatment, and each biological replicate had four technical repeats. The relative gene expression levels were calculated using the 2^−ΔΔCT^ method [34].

## 3. Results

### 3.1. Genetic Characteristics of Resistance of IR77298-14-1-2::IRGC117374-1 to U. virens

**Figure 1:**
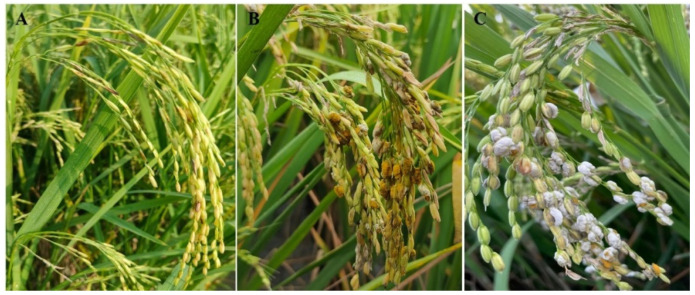
Symptoms of rice false smut in the parent and the F_1_ generation 21 d after inoculation. (**A**) Resistant parent IR77298-14-1-2::IRGC117374-1. (**B**) Susceptible parent 9311. (**C**) F1 generation.

**Figure 2:**
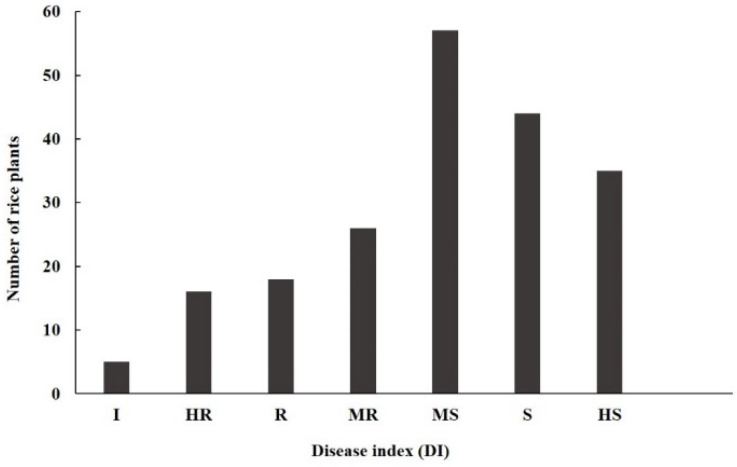
Number of rice plants in the F_2_ generation population under different resistance levels.

The cross was carried out with resistant variety IR77298-14-1-2::IRGC117374-1 as the male parent and susceptible variety 9311 as the female parent; then, the resistance of parents and filial generations was identified using strongly virulent mixed strains of *U. virens*. IR77298-14-1-2::IRGC117374-1 panicles were resistant and did not exhibit diseased grains 21 d after inoculation. In contrast, susceptible parent 9311 produced many diseased grains after inoculation, and the DI ranged from 7 to 9. All F_1_ generation plants were susceptible (S) or highly susceptible (HS), and no resistant plants were found (Figure 1), indicating that the gene of IR77298-14-1-2::IRGC117374-1 controlling rice resistance to RFS was recessively inherited. Of the 201 rice plants from the F_2_ generation population, 5 were immune (I), 16 were highly resistant (HR), 18 were resistant (R), 26 were moderately resistant (MR), 57 were moderately susceptible (MS), 44 were susceptible (S), and 35 were highly susceptible (HS) (Figure 2, Supplementary Table S3). A disease index of 20 was the dividing line between resistance and susceptibility. The number of resistant and susceptible plants was 65 and 136, respectively, and the ratio of resistant (R) and susceptible (S) separation was close to 1:2.1, which is not in line with the theoretical ratio of single-gene genetic separation (1:3). The results indicate that the resistance of IR77298-14-1-2::IRGC117374-1 to RFS was controlled by multiple loci.

### 3.2. Mapping QTL for Resistance to RFS Based on the BSA-Seq Technique

#### 3.2.1. Sequencing Data and Quality Assessment

The experiment constructed four DNA libraries (IR77298-14-1-2::IRGC117374-1 (male parent), 9311 (female parent), R-pool, and S-pool) for BSA-seq and sequenced by the Illumina NovaSeq platform. A total of 258,279,848 raw reads were generated from the samples. After data filtering, clean reads were obtained from a different sample ranging from 49.35 to 79.45 Mb. The GC content and Q30 were 44.43–44.90% and 93.59–94.05%, respectively. Compared with the reference genome of Nipponbare, more than 98.25% of clean reads in each sample were mapped to the reference sequence, with a sequencing depth between 22.64 and 25.17 folds. The one-fold coverage ratio ranged from 90.84 to 97.21% (Table 1). The results showed that the sequencing depth of each sample was sufficient, the sequencing data were of high quality, and the comparison results of the sequencing data were normal, which could be used for subsequent mutation detection and correlation analysis.

#### 3.2.2. Variation Detection of BSA-Seq Data

There are two types of SNP variations: transition (Ts) and transversion (Tv). Generally, transition is more likely to occur than transversion, so the transition/transversion (Ts/Tv) ratio is generally greater than one. Our analysis showed that the Ts/Tv values were 2.4184, 2.4129, 2.402, and 2.4069 in 9311, IR77298-14-1-2::IRGC117374-1, R-pool, and S-pool, respectively (Table 2). Furthermore, SNPs and Indels, including heterozygotes (HET) and homozygotes (HOM), were investigated. The higher the number of homozygous SNPs, the greater the difference between the sample and the reference genome. Conversely, the higher the number of heterozygous SNPs, the greater the heterozygosity of the sample. There were 2,954,310, 2,913,481, 3,482,716, and 3,504,278 SNPs in 9311, IR77298-14-1-2::IRGC117374-1, R-pool, and S-pool, respectively, among which 583,954, 614,673, 2,355,884, and 1,960,376, respectively, were heterozygotes and 2,370,356, 2,298,808, 1,126,832 and 1,543,902, respectively, were homozygotes (Table 2). The heterozygous sites of the R-pool and S-pool accounted for 67.65% and 55.94% of the total SNP sites, respectively, indicating that the difference between the F_2_ population and the reference genome was small and that the difference between the two parents and the reference genome was large.

The types of Indel mutations included insertions and deletions. A total of 462,940, 454,802, 542,475, and 543,968 Indels were identified in 9311, IR77298-14-1-2::IRGC117374-1, R-pool, and S-pool, respectively (Table 3). Among them, 227,374, 222,583, 276,475, and 271,429 Indel mutation sites, respectively, were insertions, and 257,169, 252,521, 316,251, and 309,933 Indel mutation sites, respectively, were deletions.

#### 3.2.3. Annotation of SNPs and Indels

The SNPs of the samples were annotated by ANNOVAR software [28] combined with reference genome annotation information. Our analysis results showed that the R-pool had 2,494,483 SNPs in the intron and 226,625 SNPs in the exon, including 120,014 nonsynonymous mutations, accounting for 52.96% of the exon region. S-pool had 398,256 mutations in the intron and 221,920 SNPs in the exon, including 117,726 nonsynonymous mutations, accounting for 53.09% of the exon region. There were 95,232 and 94,906 nonsynonymous mutations detected in parent 9311 and IR77298-14-1-2::IRGC117374-1, accounting for 50.99% and 50.50% of the exon region, respectively (Supplementary Table S4). In addition, we annotated and counted the Indels in each sample. In the R-pool, there were 84,192 Indels in the intron and 14,098 Indels in the exon, including 4434 frameshift, 129 stop-gain and 18 stop-loss mutations, accounting for 30.13% of exons. In the S-pool, there were 89,448 Indels in the intron and 14,921 Indels in the exon, including 4535 frameshift, 127 stop-gain, and 16 stop-loss mutations, accounting for 31.45% of exons. There were 3504 and 3531 frameshift 102 and 105 stop-gain, 14 and 12 stop-loss mutations detected in parent 9311 and IR77298-14-1-2::IRGC117374-1, respectively, accounting for 50.99% and 50.50% of the exon, respectively (Supplementary Table S5).

#### 3.2.4. Association Analysis of BSA-Seq Data

In this experiment, the Euclidean distance (ED) algorithm [29] was used to conduct an association analysis of the detected SNPs and Indels. To reduce the background noise caused by a small difference in the ED values, the ED^4^ was calculated and fitted to the ED^4^. The median of three standard deviations was chosen as the threshold, and the region higher than the threshold was taken as the candidate interval. In this study, the regions coidentified in both SNPs and Indels were used as candidate regions for the association of resistance to RFS. As shown in Supplementary Figure S1, two regions located on chromosomes 6 (NC_029261.1) and 12 (NC_029267.1) showed points exceeding the threshold value, indicating that these two chromosome regions were associated regions for resistance to RFS. Three associated regions were identified on chromosome 12, covering 0.57 Mb (10.58–11.15 Mb), 3.07 Mb (16.56–19.63 Mb), and 2.21 Mb (21.49–23.70 Mb), and including 26, 109, and 125 genes, respectively. An associated region was identified on chromosome 6, ranging from 0.03 to 0.81 Mb (0.78 Mb), containing 64 genes. In total, 324 candidate genes were identified in these candidate regions (Table 4).

### 3.3. QTL Identification for RFS Resistance Based on SSR Markers

#### 3.3.1. Screening of SSR Molecular Markers for Resistance to RFS

**Figure 3:**
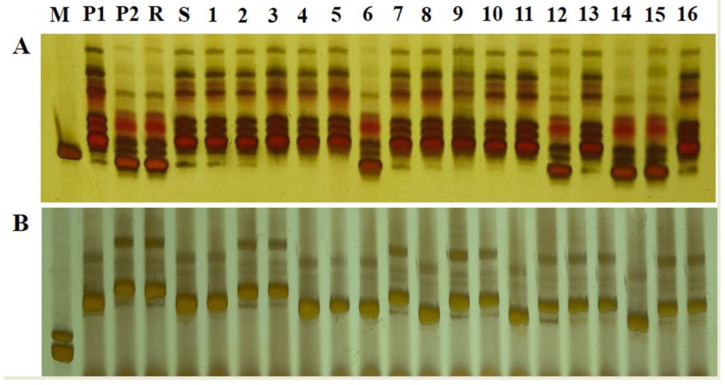
Amplification results of markers linked to a resistance site using bulk segregant analysis and some single plants from the F_2_ population. (**A**) RM28195. (**B**) RM5341. Note: M: marker; P1: susceptible parent 9311; P2: resistant parent IR77298-14-1-2::IRGC117374-1; R: resistant bulk; S: susceptible bulk; 1–16: individual plants from the F_2_ population.

In this study, 871 SSR markers, which were evenly distributed on 12 rice chromosomes, were used to test DNA polymorphism in resistant parent IR77298-14-1-2::IRGC117374-1 and susceptible parent 9311. We identified 71 SSR markers with polymorphisms between parents (Supplementary Table S1), and the polymorphism rates of SSR markers on each chromosome varied between parents, ranging from 1.45 to 19.05%, with an average polymorphism rate of 8.13% (Supplementary Table S6). Among 71 primers with polymorphism between resistant and susceptible parents, RM5341 and RM28195 were polymorphic between the R-pool and the S-pool (Figure 3). According to the published rice gene database, both molecular markers were located on chromosome 12.

#### 3.3.2. Construction of Genetic Map and QTL Mapping

**Figure 4:**
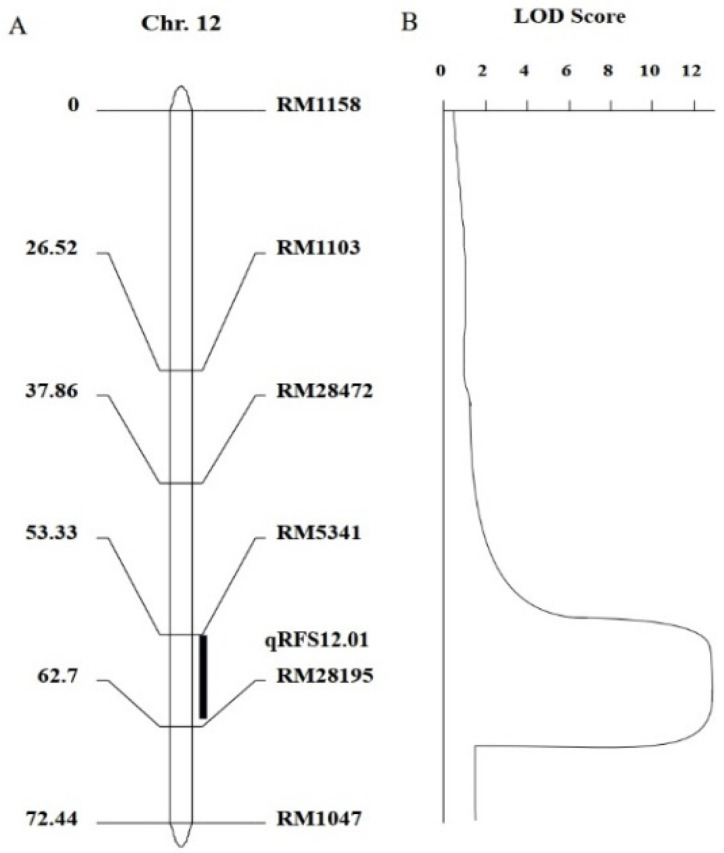
QTL detection for rice false smut resistance in the linkage map. (**A**) Linkage map of the SSR markers; (**B**) composite interval mapping of QTL.

We redesigned SSR molecular markers near RM5341 and RM28195 and screened another four polymorphic markers between parents, RM1158, RM1103, RM28472, and RM1047. These six molecular markers were linked to genes for RFS resistance to varying degrees. The genotypes of the F_2_ generation population were analyzed with the six polymorphic markers (Supplementary Table S7). We used QTL ICiMapping V4.0 software to construct linkage maps according to the genotypes of the F_2_ population (Figure 4A). Then, according to the genotype of individual plants in F_2_ population and the phenotype of resistance to RFS in the field, compound interval mapping was performed using the ICIM-ADD method in IciMapping 4.0 software to obtain QTL localization interval maps (Figure 4B). As shown in Figure 4B, there was a peak value of 12.76 between markers RM5341 and RM28195, which was greater than the LOD threshold of 3.0. Therefore, we speculated that there was a major QTL between RM5341 and RM28195, with a genetic distance of 9.37 cM. The identified QTL was named *qRFS12.01*. According to the physical location of SSR markers on chromosomes, the mapped QTL was located between 18,152,114 bp and 19,155,838 bp on chromosome 12. In addition, QTL *qRFS12.01* flanked by RM5341 and RM28195, had the greatest effect on resistance to RFS, explaining 28.74% of the phenotypic variance, indicating that the two markers could be used for marker-assisted selection of RFS resistance derived from IR77298-14-1-2::IRGC117374-1.

### 3.4. Identification of Candidate Genes in Final Association Regions

**Figure 5:**
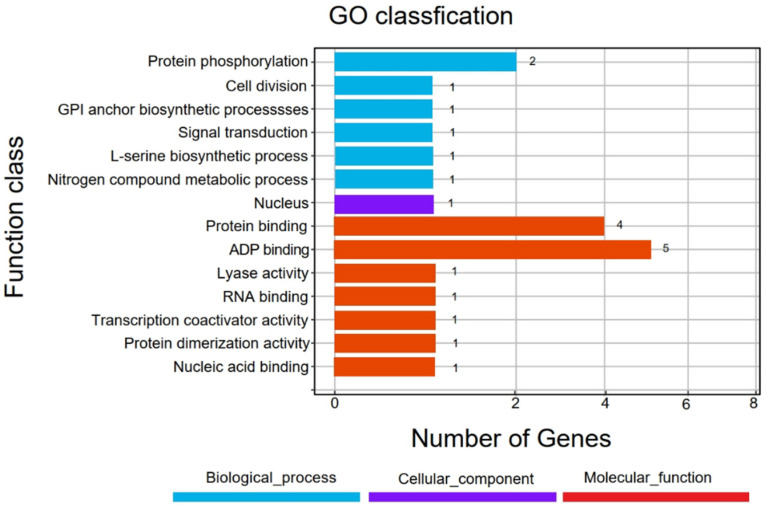
GO annotation of genes in candidate regions.

The combination of BSA-seq and SSR marker localization suggested that there were overlapping regions on chromosome 12 of the associated regions. To more accurately explore the genes related to RFS resistance, the overlapping region was selected as the final candidate region. The physical distance of the overlapping interval was 18.152–19.156 Mb, and the size of the region was 1.004 Mb, which was the region between SSR markers RM28195 and RM5341 (*qRFS12.01*), and contained 41 candidate genes, 22 of which were successfully annotated. These annotated genes fell into three main categories: biological processes, molecular functions, and cellular components (Figure 5).

The first category includes genes primarily involved in biological processes, such as protein phosphorylation (GO: 0006468), cell division (GO: 0051301), GPI anchor biosynthetic process (GO: 0006506), signal transduction (GO: 0007165), L-serine biosynthetic process nitrogen compound metabolic process (GO: 0006564), and nitrogen compound metabolic process (GO: 0006807). Infection with *U. virens* is biological stress that can induce plant signal transduction, protein phosphorylation, nitrogen metabolism, and other biological processes to achieve pathogenicity. According to the bioinformatics function and expression site of rice gene annotation, six genes were predicted to be related to RFS resistance, including *LOC_Os12g30570*, *LOC_Os12g31450*, *LOC_Os12g31480*, *LOC_Os12g31640*, *LOC_Os12g31820*, and *LOC_Os12g31830* (Supplementary Table S8).

The second category included genes encoding molecular functions, such as protein binding (GO: 0005515), ADP binding (GO: 0043531), lyase activity (GO: 0016829), RNA binding (GO: 0003723), transcription coactivator activity (GO: 0003713), protein dimerization activity (GO: 0046983), and nucleic acid binding (GO: 0003676). To resist pathogen invasion, rice has formed an extremely complex defense system in which transcription factors, plant disease resistance genes, and catalytic active enzymes participate in disease resistance. Therefore, it was predicted that these genes may be involved in the regulation of RFS resistance, including *LOC_Os12g30540*, *LOC_Os12g30590*, *LOC_Os12g30610*, *LOC_Os12g30760*, *LOC_Os12g30824*, *LOC_Os12g30920*, *LOC_Os12g31000*, *LOC_Os12g31160*, *LOC_Os12g31200*, *LOC_Os12g31340*, *LOC_Os12g31350*, *LOC_Os12g31620*, *LOC_Os12g31748*, and *LOC_Os12g31800* (Supplementary Table S8).

In summary, six genes (*LOC_Os12g30570*, *LOC_Os12g30590*, *LOC_Os12g31160*, *LOC_Os12g30760*, *LOC_Os12g31200*, and *LOC_Os12g31620*) were closely related to RFS resistance according to further verification of the rice genome annotation database (http://rice.uga.edu/index.shtml (accessed on 26 June 2023)), and these genes were highly expressed in leaves, inflorescence, pistil and anther. Of these candidate genes, five genes (*LOC_Os12g30590*, *LOC_Os12g30760*, *LOC_Os12g31160*, *LOC_Os12g31200*, and *LOC_Os12g31620*) encoded disease resistance proteins, namely RGA2, At4g10780, Pik-2-like, RGA4-like, and RPM1 containing an NB-ARC domain-containing protein, respectively. *LOC_Os12g30570* encodes a serine/threonine-protein kinase STY13-like (Table 5). Therefore, we preliminarily speculated that these six genes were most correlated with rice resistance to RFS.

### 3.5. Expression Profile Analysis of the Candidate Genes

**Figure 6:**
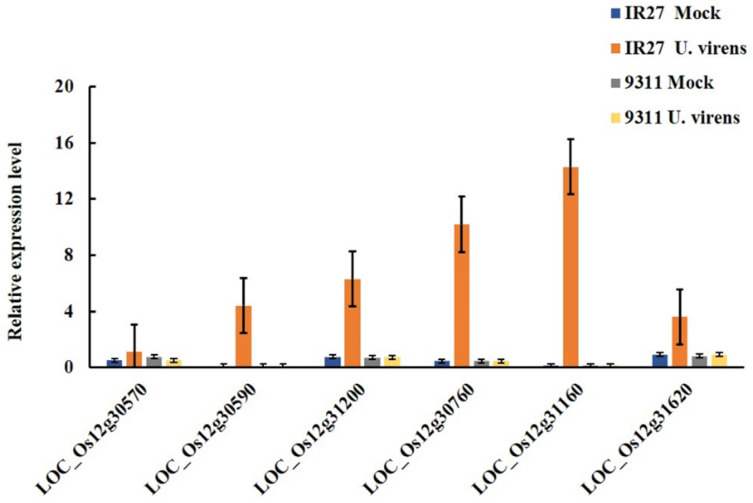
qRT-PCR analysis of the relative expression level of the predicted candidate genes located in the mapping region.

We further performed an RT-PCR assay to determine the expression profiles of the six candidate genes between infection of *U. virens* and mock inoculation in resistant parent IR77298-14-1-2::IRGC117374-1 and susceptible parent 9311. The analysis identified six candidate genes with a response to the infection of *U. virens*, five of which (*LOC_Os12g30590*, *LOC_Os12g31200*, *LOC_Os12g30760*, *LOC_Os12g31160*, and *LOC_Os12g31620*) were significantly induced by inoculation with *U. virens* in resistant parent IR77298-14-1-2::IRGC117374-1 (Figure 6). Therefore, we speculate that these five genes are closely related to resistance to RFS.

## 4. Discussion

RFS is a rice disease with a wide incidence area that seriously affects the yield of rice after rice blast, rice sheath blight, and rice bacterial leaf blight [35]. Disease control is an important way to reduce rice yield loss. At present, the prevention and control measures of RFS mainly rely on fungicides. However, overreliance on fungicides will cause pathogens to develop resistance and pollute the environment [7]. Therefore, there is an urgent need for efficient, environmentally friendly, and economical disease management strategies. Breeding and planting disease-resistant cultivars are considered an economical and effective measure for reducing the damage caused by RFS. However, due to the late start of research on the inheritance of resistance to RFS and the molecular mechanisms of rice resistance to RFS, there are still few germplasm resources for resistance to RFS disease. IR77298-14-1-2::IRGC117374-1 is a highly resistant rice material identified from early germplasm resources [23], but the inheritance mechanism of resistance is still unclear, and there are few reports about resistance genes in rice at home and abroad. In this study, we mapped the resistance QTL for RFS from the highly field-resistant rice IR77298-14-1-2::IRGC117374-1. This finding holds great value in elucidating the molecular basis of rice resistance to RFS.

BSA-seq has been widely used to map the QTL of target traits in crop species [18,36]. Some studies have reported that BSA-seq can be combined with other techniques to further reveal key functional candidate genes, such as with RNA-Seq, BSR-seq, and the linkage mapping approach [17,18,20]. In this study, the combination of BSA-seq and SSR markers was used for the first time to identify QTL conferring resistance to RFS. First, BSA-Seq association analysis identified four RFS resistance QTLs, three of which were distributed on chromosome 12 and one on chromosome 6. One QTL for resistance to RFS was mapped on chromosome 12 based on SSR molecular markers. Interestingly, the results of the two QTL mapping approaches were compared, and there was an overlapping region on chromosome 12; thus, this region was used as a candidate region for resistance genes of RFS disease. By combining the results of the two QTL mapping methods, this study improved the accuracy of QTL mapping for rice resistance to RFS disease and narrowed the candidate regions of target genes, which is of great significance for the mapping and cloning of important resistance genes in rice.

To date, more than 30 RFS-resistance QTLs have been identified in biparental populations crossed with resistant and susceptible varieties [12,16]. This experiment investigated resistance to RFS in an F_2_ population from a cross between resistant parent IR77298-14-1-2::IRGC117374-1 and highly susceptible parent 9311. Based on two QTL mapping approaches, one QTL (*qRFS12.01*) was mapped to chromosome 12. We also compared our mapping results with previous reports and found that *qRFS12.01* is a novel resistance locus and has no overlap with previous results. Moreover, *qRFS12.01* explained more than 28% of the phenotypic variance in RFS resistance QTLs. Researchers have previously reported that a major quantitative locus *qFsr1*, *qFsr8–1*, from MR183-2 explained 26.0% of phenotypic variance [12]. Therefore, it is promising to transfer these major resistance QTL to rice varieties with good agronomic traits to improve resistance to RFS.

Thus far, very few RFS resistance QTLs have been finely mapped, revealing the presence of resistance genes (R genes) [11,15,16]. Previous research has identified an RFS-resistance candidate gene encoding DNA methylase 2 on chromosome 1 of the resistant line Nanjing11 [11]. More recently, several candidate RFS-resistance loci have been fine-mapped in the RFS-resistant line RYT2668, such as *qRFSr4.3* harboring leucine-rich repeat (LRR) receptor protein kinase, *qRFSr9.1* comprising four disease resistance genes, and *qRFSr11.1* encoding RPM1 and NBS-LRR domain-containing proteins [15]. In this study, we mapped a major locus in RFS-resistant line IR77298-14-1-2::IRGC117374-1, which helped to identify a large number of putative resistance genes. Based on gene prediction and annotation in the region of major QTL *qRFS12.01*, several genes involved in RFS resistance were predicted. For instance, five genes, namely *LOC_Os12g30590*, *LOC_Os12g30760*, *LOC_Os12g31160*, *LOC_Os12g31200*, and *LOC_Os12g31620* encode disease resistance proteins RGA2, At4g10780, Pik-2-like, RGA4-like, and RPM1 containing NB-ARC domain containing proteins, respectively. Previous studies have shown that NB-ARC is a main structural component of the NBS-LRR type of plant disease resistance protein. The NB-ARC domain interacts with nucleic acid to regulate signal transduction pathways in antidisease reactions [37,38]. RGAs represent a large class of potential *R*-genes in plants and are characterized by conserved domains and structural features, making them candidate genes for disease resistance [39,40]. It has been reported that the *Pik* locus, which contains many rice blast *R* genes, has attracted considerable interest and has been used in numerous rice breeding programs [41,42]. RPM1 belongs to the class of CC-NBS-LRR protein kinase, which plays a positive regulatory role in the disease resistance system against rice blast and bacterial leaf blight [43,44]. Therefore, it is possible that these genes play an important role in RFS resistance. However, further validation is required to confirm their function in providing disease resistance.

## 5. Conclusions

In conclusion, we identified a novel QTL *qRFS12.01* for RFS resistance in IR77298-14-1-2::IRGC117374-1 by combining BSA-seq and SSR marker mapping approaches. The *qRFS12.01*-linked SSR markers can be used in a marker-assisted selection of cultivars resistant to RFS. Some candidate genes referring to RFS resistance were predicated in this QTL region. It is necessary to study the mechanism of these candidate genes in rice further. These findings provide important information for cloning the genetic loci controlling false smut resistance and for developing rice cultivars with false smut resistance.

## Figures and Tables

**Table 1:** Sequencing data statistics for BSA-seq samples.

Sample	Raw Reads	Clean Reads	Mapped Rate (%)	Q30 (%)	GC Content (%)	Average Depth	Coverage Ratio 1× (%)
9311	49,872,668	49,349,016	98.25%	93.95	44.43	23.3	91.13
IR77298-14-1-2::IRGC117374-1	52,778,094	50,273,734	98.45%	94.05	44.81	22.64	95.57
R-pool	79,854,494	79,044,776	98.52%	93.59	44.9	23.37	97.21
S-pool	75,774,592	75,139,598	98.52%	93.96	44.63	25.19	90.84
Total	258,279,848	253,807,124					

**Table 2:** Genotype statistics for each sample in a population (SNPs).

Sample	Total SNPs	HET	HOM_ALT	Het Rate	Ts	Tv	Ts/Tv
9311	2,954,310	583,954	2,370,356	19.77%	2,184,841	903,425	2.4184
IR77298-14-1-2::IRGC117374-1	2,913,481	614,673	2,298,808	21.10%	2,156,436	893,710	2.4129
R-pool	3,482,716	2,355,884	1,126,832	67.65%	2,637,543	1,098,054	2.402
S-pool	3,504,278	1,960,376	1,543,902	55.94%	2,699,874	1,121,715	2.4069

**Table 3:** Genotype statistics for each sample in a population (Indels).

Sample	Total Indels	HET	HOM_ALT	Insertions	Deletions
9311	462,940	66,304	396,636	227,374	257,169
IR77298-14-1-2::IRGC117374-1	454,802	71,566	383,236	222,583	252,521
R-pool	542,475	360,446	182,029	276,475	316,251
S-pool	543,968	290,830	253,138	271,429	309,933

**Table 4:** Candidate regions for resistance identified by BSA-seq association analysis.

Chromosome	Start-Position	End-Position	Size (Mb)	Number of Gene
12 (NC_029267.1)	10,575,821	11,148,578	0.57	26
12 (NC_029267.1)	16,557,521	19,631,478	3.07	109
12 (NC_029267.1)	21,494,927	23,703,456	2.21	125
6 (NC_029261.1)	25,365	805,366	0.78	64
Total	—	—	6.63	324

**Table 5:** Candidate genes for resistance to rice false smut.

Gene ID	Protein Length	Gene Product Name	Expression Site
*LOC_Os12g30570*	385	Serine/threonine-protein kinase STY13-like, partial	Pre-emergence inflorescence, leaves, anther, pistil
*LOC_Os12g30590*	426	Disease resistance protein RGA2, NB-ARC domain-containing protein, expressed	Leaves, inflorescence, anther, pistil, endosperm, shoots
*LOC_Os12g30760*	1056	Putative disease resistance protein At4g10780, NB-ARC domain-containing protein	Leaves, inflorescence, anther, pistil
*LOC_Os12g31160*	936	Disease resistance protein Pik-2-like, NB-ARC domain-containing protein, expressed	Leaves, inflorescence, anther, pistil
*LOC_Os12g31200*	1009	Disease resistance protein RGA4-like, NB-ARC domain-containing protein, expressed	Leaves, inflorescence, anther, pistil
*LOC_Os12g31620*	902	Disease resistance protein RPM1, NB-ARC domain-containing protein, expressed	Leaves, inflorescence, anther, pistil

## Data Availability

Data are contained within the article.

## Supplementary Materials

The following supporting information can be downloaded at: https://www.mdpi.com/article/10.3390/biom14010079/s1. Supplementary Table S1. SSR markers with polymorphisms in the tested rice. Supplementary Table S2. qRT-PCR primers used in this study to analyze candidate resistance genes. Supplementary Table S3. Disease index of individual rice plants in the F2 generation population. Supplementary Table S4. Annotated statistics of population SNPs. Supplementary Table S5. Annotated statistics of population Indels. Supplementary Table S6. Polymorphism marker screening statistics among parents. Supplementary Table S7. Genotype analysis of the F2 population. Supplementary Table S8. Statistical analysis of resistance candidate genes. Supplementary Figure S1. Calculation of ED4 values on the chromosome to identify the candidate regions associated with resistance to RFS based on BSA-seq.
