# Supplementary figures and images for: Conjunctive Analysis of BSA-Seq and SSR Markers Unveil the Candidate Genes for Resistance to Rice False Smut
Source: Biomolecules. 2024 Jan 8;14(1):79. doi: 10.3390/biom14010079 (PMC10813778; doi:10.3390/biom14010079)

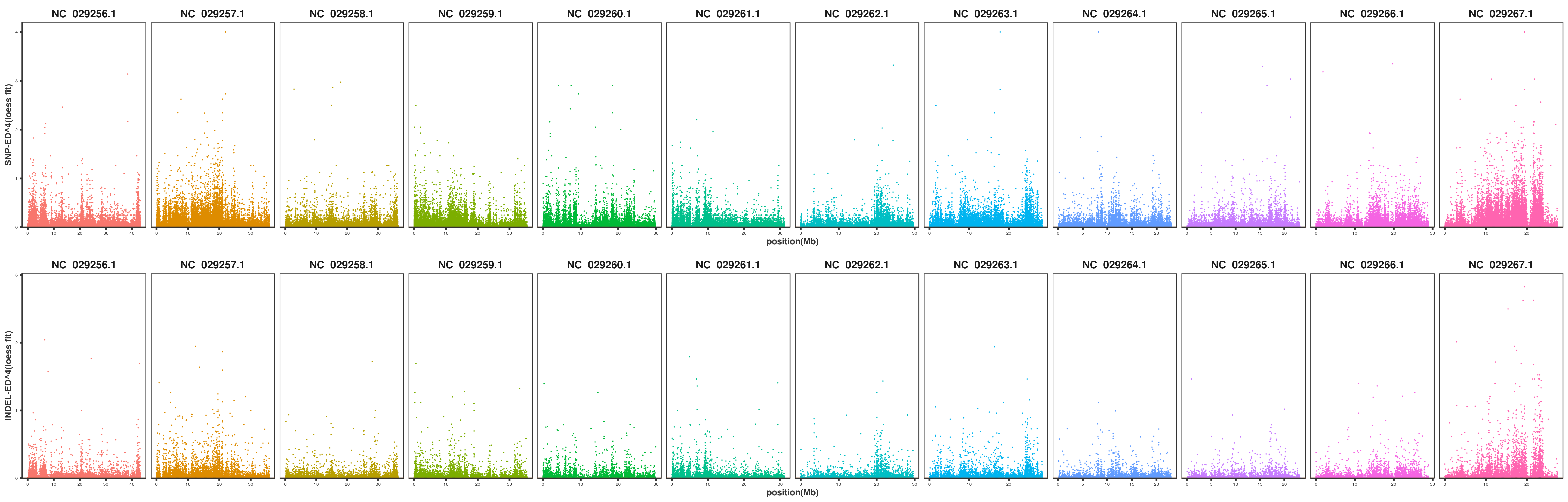

Supplement: Supplementary file 1 [file biomolecules-14-00079-s001.zip › Supplementary Figure S1.jpg]
